# Episodic Neuropathic-Like Musculoskeletal Pain Associated With Ritlecitinib Therapy in Alopecia Universalis: A Case Report

**DOI:** 10.7759/cureus.94571

**Published:** 2025-10-14

**Authors:** Sumeet Bhardwaj, Sasank Aramandla, Affan Naveed, Kevin Tu, Thinh D Mai

**Affiliations:** 1 Psychiatry, Kansas City University of Medicine and Biosciences, Kansas City, USA; 2 Internal Medicine, Kansas City University of Medicine and Biosciences, Kansas City, USA; 3 Radiology, Kansas City University of Medicine and Biosciences, Kansas City, USA; 4 Basic Medical Sciences, Kansas City University of Medicine and Biosciences, Kansas City, USA

**Keywords:** alopecia areata, alopecia universalis, autoimmune disease, cyp2c9, deuruxolitinib, jak inhibitors, neuropathic pain, pharmacogenomics, ritlecitinib, treatment resistance

## Abstract

This case report describes a 30-year-old male patient with extensively treatment-resistant alopecia universalis in the context of autoimmune and atopic comorbidities, including eczema, asthma, severe allergic reactions, and Hashimoto’s thyroiditis. The Janus kinase (JAK) inhibitors described in this case report are considered treatment failures, defined as the absence of clinically meaningful hair regrowth after at least six months of therapy or discontinuation due to adverse effects. Despite the recent approval of JAK inhibitors for severe alopecia areata, our patient experienced treatment failure with multiple agents and, while on ritlecitinib, developed a debilitating, episodic musculoskeletal pain syndrome. To our knowledge, such a presentation has not been well characterized in the literature. While causality cannot be firmly established, this case raises the possibility of a novel treatment-related adverse effect that may significantly impair quality of life. These findings underscore the importance of ongoing pharmacovigilance as JAK inhibitors are increasingly utilized in alopecia management.

## Introduction

Alopecia areata (AA) is regarded as a cell-mediated autoimmune disorder that involves the loss of immunoprivilege among melanin-producing anagen hair bulbs. Patients are otherwise generally healthy. In rare cases, AA progresses to total body hair loss, known as alopecia universalis (AU). AA is classified as a "type 1 inflammatory" disorder [1]. Type 1 inflammation typically involves targeting intracellular pathogens such as viruses and specific forms of bacteria, and the activation of Th1 cells, CD8+ T cells, and macrophages [1]. Reviews further highlight that dysregulation of the Janus kinase-signal transducer and activator of transcription (JAK-STAT) signaling pathway and downstream interferon responses represent central drivers of AA pathogenesis [2]. Extensive evidence in mouse models provides sufficient support that IFN-γ plays a key role in the pathogenesis of AA [3]. Studies in humans also demonstrate higher levels of IFN-γ within the serum of patients with AA compared to controls [3]. IFN-γ-producing cells are detected more frequently in the perifollicular infiltrate of AA lesions than in healthy skin [3]. In addition, other factors attributed to the pathogenesis of AA involve abnormalities in the regulation of major histocompatibility complex (MHC) class I in the proximal outer root sheath and matrix cells, where it is downregulated [4].

Interferon-γ is produced by autoreactive CD8+ NKG2D+ T cells and innate lymphoid cells, and this cytokine environment in the local tissue leads to upregulation of MHC class I/II molecules on hair follicle keratinocytes. When these molecules are presented on the keratinocyte cell membrane, they become susceptible to immune-mediated attack by additional cytotoxic T cells [4]. This drives cytokines like CXCL9, CXCL10, and CXCL11. These chemokines are also known to activate the type 1 response, which recruits cytotoxic CD8 T cells to the region to attack the keratinocytes [5]. Phase II clinical trials demonstrate that ruxolitinib, which inhibits JAK-STAT pathways, can reverse AA. However, there is no phase III or regulatory approval for this indication.

As AA is characterized as an autoimmune process that can be reversed, as long as the hair follicles are not completely destroyed, there remains a possibility of hair regrowth if the autoimmune process is controlled [6]. Prat et al. state that AA is reversible by Janus kinase (JAK) inhibitors and that hair growth can be restored by suppressing IFN-γ signaling and its downstream effects [6]. This is a genome-wide association study that assesses various pathways that may be targeted to reverse AA.

Other proposed mechanisms for AA include a CTLA-4 gene transcriptome defect that causes misfolding in the protein responsible for CD4 helper T cells to modulate cytotoxic T cells and promote tolerance of the keratinocyte cellular environment [7]. This pathway can be targeted by recombinant CTLA-4 immunoglobulin to treat AA [8].

AA is characterized in the literature as a primarily autoimmune process. The disease requires a combination of genetic predisposition to autoimmune diseases as well as environmental triggers that drive the pathogenesis of the condition. The MHC I and II haplotypes that are primarily discussed represent the first requirement; then environmental triggers are necessary to drive the pathogenesis of this condition. These include psychological stress, smoking, alcohol consumption, decreased sleep, obesity, fatty acid intake, and gluten consumption [9].

## Case presentation

We report a longitudinal case of a 30-year-old male third-year medical student with AU that demonstrates resistance to most standard treatment regimens. The patient had an extensive family history of autoimmune disease, including eczema, asthma, arthritis of unknown etiology, hypothyroidism of unclear etiology, and multiple severe allergic reactions. This background provides insight into the likely genetic and immunologic origins of his AU and may contribute to its resistance to treatment.

The patient’s AU emerged at seven years of age, preceded by burning and tingling sensations shortly before initial hair loss. Hair loss progressed rapidly over several weeks, with nail abnormalities such as onychorrhexis appearing later. After dermatologic assessment, at age 11, he began clobetasol propionate 0.05% (applied twice daily for three to six months), which failed to produce meaningful clinical improvement. Intralesional corticosteroids such as triamcinolone acetonide, typically administered at 2.5-5 mg/mL in 0.1 mL per site every 3-6 weeks, are not used due to their traditional application in patchy A and limited efficacy in alopecia universalis. During this period, he also used topical minoxidil 5% foam twice daily for six months without benefit. At age 12, anthralin 0.5-1% cream was introduced as a short-contact regimen (20-60 minutes daily before rinsing), but this also failed to achieve clinical improvement.

Anthralin is selected in part due to evidence suggesting that local inflammation may distract the immune system from attacking hair follicles. Despite several months of consistent use, no regrowth was observed, and therapy was discontinued. At age 12, after multiple treatment failures, he underwent topical contact immunotherapy with squaric acid dibutyl ester (SADBE), initiated at 0.001% and titrated up to 2% as tolerated. Weekly applications are performed under occlusion for 24-48 hours, but after more than six months, no clinically significant regrowth occurs. Treatment was further complicated by painful blistering, pruritus, and pigmentary changes, which led to discontinuation.

The patient was understandably disappointed, and treatment options became increasingly limited. Systemic corticosteroids and immunosuppressants, including methotrexate, cyclosporine, azathioprine, and mycophenolate mofetil, were discussed, but he declined due to the risks of adverse effects such as infection and growth suppression at his young age. Ultimately, all therapies were discontinued as they proved ineffective.

After many years without intervention, at age 28, his dermatologist revisited treatment options and discussed JAK kinase inhibitors. At age 29, he began tofacitinib 5 mg twice daily, later transitioning to ritlecitinib 50 mg once daily, each administered for six months as recommended for severe AA. After six months of therapy, tofacitinib failed to produce substantial regrowth in any body area and was subsequently discontinued. Topical ruxolitinib was not considered due to its limited efficacy in severe AA.

During treatment with ritlecitinib, the patient developed episodic, asymmetric musculoskeletal pain involving the left buttock, left thigh, and left calf, with greatest severity in the right shoulder. The pain never occurred bilaterally. It was localized but with intermittent radiation, described qualitatively as an intense gnawing or burning sensation. On a subjective pain scale, the patient rated the intensity at approximately 7 out of 10 during exacerbations. When the right arm was affected, the pain localized to the forearm and the antecubital area, with radiation between these sites and extending back up to the shoulder. Movement of the arm sometimes exacerbates the pain. The patient reported compulsions to strike, scratch, or massage the affected region to obtain transient relief, which were most intense in the right shoulder, underscoring the neuropathic character of the symptoms.

The precise onset was somewhat unclear but occurred several months after initiating ritlecitinib. Episodes arose unpredictably after dosing, remained constant once initiated, and radiated in a nerve-like distribution down the affected limb. They typically lasted up to a full day and were frequently followed by localized tenderness, swelling, warmth, and erythema. The pain significantly impacted activities of daily living; for instance, when present in the right arm, it made driving with that arm difficult and uncomfortable. Despite these features, no numbness, tingling, weakness, or loss of range of motion was reported. The pain in the right shoulder was self-reported by the patient to have lasted for a week. These symptoms that arose during ritlecitinib therapy may represent an adverse drug reaction, though causality cannot be firmly established without further evaluation.

Symptomatic relief was attempted with lidocaine patches and ibuprofen, with moderate benefit, but his quality of life remained significantly impaired. He reported the adverse effect to his dermatologist and was evaluated for rhabdomyolysis, with serum creatine kinase levels found to be normal. Based on the clinical context, the dermatologist did not consider neurologic or orthopedic referral necessary and instead recommended discontinuation of ritlecitinib, documenting the incident. The dermatologist recommended discontinuing ritlecitinib due to the same substantial lack of regrowth as tofacitinib on any body region, as well as the emergence of adverse effects. The absence of neurologic deficits, trauma, or alternative causes, combined with the tightly time-linked onset after dosing, supports a causal association with ritlecitinib. Following discontinuation, the pain recurred intermittently for several months before resolving completely.

**Figure 1:**
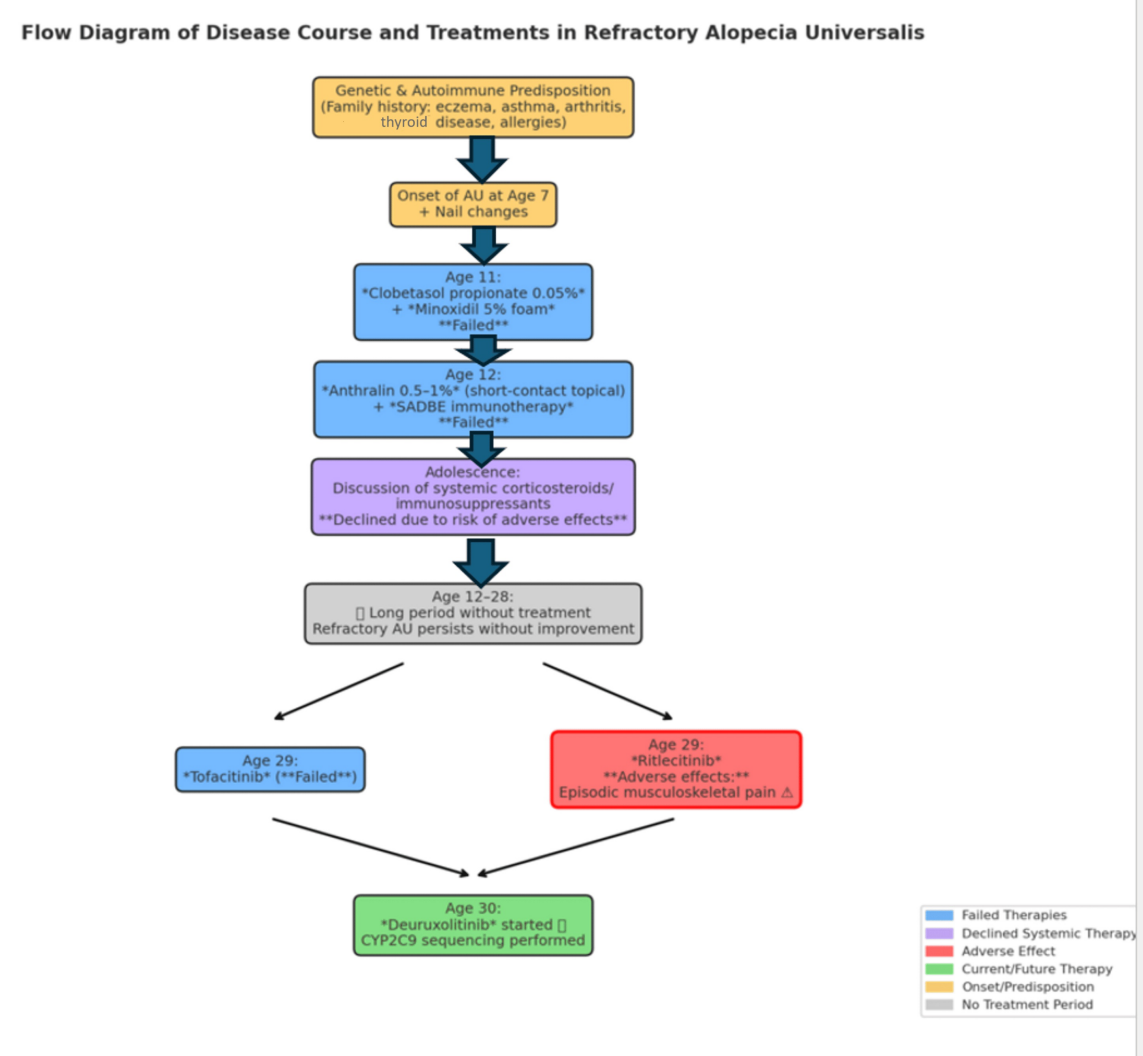
Flow Diagram of the Clinical Course and Treatment Decisions in Refractory Alopecia Universalis 🟦 Blue: Failed therapies (topical and systemic) 🟪 Purple: Systemic corticosteroids/immunosuppressants discussed but declined due to risk of adverse effects 🟥 Red (bold border): Adverse effect (Ritlecitinib → episodic musculoskeletal pain) 🟩 Green: Current/Future therapy (Deuruxolitinib with CYP2C9 sequencing) 🟨 Amber: Onset/Predisposition (genetic, autoimmune, family history) ⬜ Gray: Period without treatment (Refractory AU persists without improvement) Arrows: Sequence of clinical events and treatment decisions AU: Alopecia Universalis; SADBE: Squaric Acid Dibutyl Ester

At age 30, following the failure of prior JAK inhibitors, the patient was approved for deuruxolitinib, a JAK1/2 inhibitor recently FDA-approved for severe AA. As part of pretreatment evaluation, CYP2C9 genotype sequencing is performed via RT-PCR at a reference laboratory, given that the drug’s metabolism is significantly influenced by CYP2C9 activity. He was identified as an intermediate metabolizer, qualifying for treatment initiation with dose adjustments and monitoring. This pharmacogenomic step underscores the importance of individualized therapy in AU. At the time of reporting, he has not yet initiated deuruxolitinib therapy, though treatment is anticipated to provide meaningful improvement. No original clinical images are available, which is transparently noted by the authors. Figure 1 illustrates the progression of the patient's alopecia and the treatments administered throughout the course.

## Discussion

A comprehensive summary of the patient’s treatment regimens, including standard dosing, duration, and clinical outcomes that were discussed, is presented in Table 1.

**Table 1:** Summary of Standard Therapies and Investigational JAK Inhibitors for Alopecia Universalis Standard dosing information is adapted from consensus statements and pivotal clinical trials [10-14]. Patient regimens and outcomes are derived from the present case. JAK: Janus kinase

Medication/Therapy	Class/Mechanism	Standard Dosing (per Dermatology Literature/Practice)	Standard Dosing (per FDA/Clinical Trials)	Patient Regimen	Duration	Outcome/Notes
Clobetasol propionate 0.05% (topical)	High-potency corticosteroid	Apply thin film BID; typical course 6–12 weeks, extendable to 3–6 months [10]	Not FDA-approved; consensus/practice-based [10]	BID	3–6 months	No meaningful regrowth
Anthralin 0.5–1% (short-contact topical)	Irritant; induces local inflammation	0.5–1% cream applied daily for 20–60 min then rinsed [10].	Not FDA-approved; consensus/practice-based [10]	Daily (20–60 min)	Several months	No regrowth; discontinued
SADBE (Squaric acid dibutyl ester, topical immunotherapy)	Contact immunotherapy; induces a local allergic reaction	Sensitization with 2% once, then 0.001–2% weekly under occlusion 24–48h [10].	Not FDA-approved; consensus/practice-based [10]	0.001% → titrated to 2% weekly under occlusion	>6 months	No regrowth; blistering, pruritus, pigment changes → discontinued
Minoxidil 5% foam (topical, OTC)	Vasodilator; prolongs anagen phase	1 mL BID to the affected scalp [11].	FDA approval for androgenetic alopecia: 5% BID [11]	BID	6 months	No response
Tofacitinib (oral)	JAK1/3 inhibitor	5 mg BID; escalation to 10 mg BID in non-responders (off-label) [12].	Not FDA-approved; open-label pilot study used 5 mg BID [12]	5 mg BID	6 months	No regrowth
Ritlecitinib (oral, Litfulo®)	JAK3/TEC inhibitor	50 mg QD consensus [13].	FDA-approved: ALLEGRO trial: 200 mg QD × 4 wk → 50 mg QD [13].	50 mg QD	6 months	No regrowth; novel AE: episodic asymmetric neuropathic-like musculoskeletal pain; discontinued
Deuruxolitinib (oral, Leqselvi®)	JAK1/2 inhibitor	8–12 mg BID investigational [14].	FDA-approved: 8 mg BID; THRIVE-AA1 tested 8 mg BID and 12 mg BID [14].	Adjusted for CYP2C9 intermediate metabolizer (dosage not determined)	Planned	Not initiated at the time of reporting

Multidimensional assessment of disease severity in alopecia universalis

While the extent of hair loss is often emphasized in AA severity, a more comprehensive evaluation incorporates additional domains. King et al. propose a multidimensional framework that considers age of onset, comorbidities, treatment resistance, and impact on quality of life [15]. Our patient developed AU at age 7, an early onset that is strongly associated with more severe and refractory disease. Although it is self-reported by the patient that signs of alopecia may have been present during infancy, hair shedding in newborns is common and typically benign, making this uncertain. His medical history, however, demonstrates significant systemic immune dysregulation. The patient's medical history documents eczema in childhood and adolescence that interferes with sleep due to incessant scratching, recurrent asthma exacerbations and severe allergies causing at least three episodes of anaphylaxis. His immune dysregulation is further highlighted by the development of Hashimoto’s thyroiditis at the age of 29, confirmed biochemically with markedly elevated anti-thyroid peroxidase antibodies indicated at 4200 IU/ml, further supporting a longitudinal autoimmune predisposition and raising concern for additional autoimmune disease in the future. Polyautoimmunity, defined as the coexistence of two or more autoimmune disorders in a single patient, was well documented in association with AA and was thought to share overlapping genetic and immunopathogenic mechanisms, including CTLA-4 polymorphisms and HLA class II associations [7,8]. This clustering warranted clinicians to maintain vigilance for other autoimmune manifestations in patients with severe AA.

Despite trials of clobetasol propionate 0.05%, minoxidil 5% foam, anthralin, squaric acid dibutyl ester (SADBE), and JAK inhibitors, he failed to achieve meaningful regrowth, with some therapies themselves contributing to morbidity, including painful blistering from SADBE and severe musculoskeletal pain from ritlecitinib. The psychosocial impact is equally profound: beginning in adolescence, he experienced prolonged social isolation, avoidance behaviors, and a cumulative psychological toll manifesting as self-doubt, anxiety, and depression. While alopecia does not interfere with his academic progress or career planning as a medical student, this history of isolation may make aspects of clinical duties, such as patient interaction and confidence in professional identity, more challenging. Taken together, his early onset, extensive comorbidities, treatment refractoriness, treatment-related morbidity, and psychosocial burden illustrate that disease severity in AU extends far beyond visible hair loss, underscoring the need for individualized, multidimensional approaches to management.

The introduction of JAK inhibitors represented a paradigm shift in AU management, supported by a robust mechanistic rationale. Aberrant activation of the JAK-STAT pathway and interferon signaling were central drivers of AA pathogenesis, and JAK inhibitors such as tofacitinib, ritlecitinib, and deuruxolitinib demonstrated promising efficacy in clinical trials [2,5]. However, this case demonstrated that even advanced targeted therapies could fail, emphasizing the heterogeneity of treatment response. Notably, the patient experienced a potentially undocumented adverse event while on ritlecitinib, characterized by episodic burning neuropathic pain. While JAK inhibitors were generally well tolerated, emerging evidence suggested their pleiotropic role in modulating neuroinflammatory signaling [16], raising the possibility that off-target effects or cytokine rebound phenomena contributed to neuropathic pain syndromes. The absence of elevated creatine kinase and preserved motor function excluded rhabdomyolysis or overt myopathy, reinforcing a neuropathic mechanism as the most plausible explanation. The symptoms observed in this case may represent an unusual or previously undocumented adverse drug reaction to ritlecitinib, though definitive causality cannot be confirmed in the absence of neurologic evaluation.

The decision to initiate deuruxolitinib in this patient was supported by recent comparative data. A 2024 network meta-analysis found that deuruxolitinib 12 mg had the highest probability of achieving clinically significant regrowth among currently available JAK inhibitors [17]. Nonetheless, given the patient’s prior adverse reaction, careful monitoring was warranted. Furthermore, pretreatment pharmacogenomic assessment of CYP2C9 genotype represented an important advancement in personalized medicine, mitigating the risk of drug accumulation and toxicity.

This case also highlighted broader implications for patient quality of life and psychosocial burden. The patient’s early-onset AU and rapid progression also aligned with prior observations that childhood-onset disease was associated with more severe phenotypes, nail changes, and poor response to conventional therapies [18]. AU, particularly when treatment-resistant, imposed profound psychological distress, often exacerbated by the unpredictability of treatment outcomes and limited therapeutic success [19]. 

The lack of regrowth despite multiple lines of treatment, including high-potency topical corticosteroids, anthralin, minoxidil, and topical immunotherapy, highlighted the limitations of localized interventions in extensive disease. These findings mirrored the Cochrane review by Delamere et al., which concluded that most topical modalities had little or no benefit in AU compared with placebo [20]. The systemic immune dysregulation evident in AU often necessitated systemic therapy for meaningful improvement. Future research should have prioritized predictive biomarkers of treatment response, strategies for long-term disease control, and safety surveillance of novel agents.

Limitations

A key limitation of this report is that no neurology referral or additional diagnostic testing (such as electromyography or nerve conduction studies) was pursued. The treating dermatologist did not deem such evaluation necessary, as the clinical presentation and temporal association with ritlecitinib were considered sufficient to recommend drug discontinuation. However, this decision resulted in the loss of an opportunity to objectively document the adverse effect. Consequently, while the symptoms strongly suggest a neuropathic phenomenon, definitive causality cannot be established.

Potential Confounders

Although the patient’s symptoms arose during ritlecitinib therapy, alternative explanations cannot be fully excluded. Musculoskeletal strain, overuse, or postural factors could plausibly account for localized pain in the shoulder, buttock, or thigh, particularly in the setting of long study hours and driving. Autoimmune-related mechanisms such as arthralgia, myositis, or fibromyalgia spectrum disorders are also possible, given the patient’s history of Hashimoto’s thyroiditis, eczema, asthma, and strong family history of arthritis. Neuropathic causes, including cervical or lumbar radiculopathy, peripheral neuropathy, or entrapment syndromes, could mimic the neuropathic-like distribution reported. Endocrine and metabolic contributors, such as hypothyroidism-related myalgia, vitamin D deficiency, or electrolyte imbalances, may also play a role. Finally, coincidental background musculoskeletal pain, common in the general population, cannot be ruled out. In the absence of a neurology consultation or additional diagnostic testing, these potential confounders remain limitations in attributing causality solely to ritlecitinib.

Safety context from the ALLEGRO program

Safety data from the ALLEGRO clinical trial program provide important context for this case. In preclinical toxicology studies, ritlecitinib was associated with reversible axonal dystrophy in dogs at supratherapeutic exposures [21]. However, this finding has not translated into human populations. Across integrated analyses of phase 2 and 3 ALLEGRO trials in severe AA, neurological events were reported, albeit rarely [22,23]. However, investigators concluded that there was no evidence of progressive nerve injury or permanent damage to the nervous system. These uncommon neurological adverse events highlight that, while ritlecitinib does not appear to cause neurotoxicity in the strict sense, neurological symptoms may still occasionally arise.

Alignment with ALLEGRO safety data

The patient’s symptoms share some features with neurological events reported in the ALLEGRO clinical trial program for ritlecitinib. Uncommon neurological events such as paresthesia/dysesthesia (≈1.6%), peripheral neuropathy (≈0.2%), and sensorineural hearing loss (≈0.7%) were observed [23]. Most adverse events in the integrated ALLEGRO analyses were mild or moderate in severity and infrequently resulted in treatment discontinuation [22,23]. Trial findings suggest a possible overlap with our patient’s neuropathic-like presentation, such as burning and gnawing pain with nerve-like radiation.

Divergence from ALLEGRO findings

However, important differences distinguish this case from the ALLEGRO trial data. First, while ALLEGRO events were mild and transient, our patient’s symptoms were severe, activity-limiting, and persistent, ultimately necessitating discontinuation of therapy. Second, the pain was asymmetric and localized to specific musculoskeletal regions, the left buttock, thigh, and calf, and most notably the right shoulder and forearm, rather than generalized sensory changes. Third, the patient reported compulsions to scratch, strike, or massage the affected areas to obtain transient relief, behaviors not described in trial reports. Fourth, episodes in this case were followed by focal tenderness, swelling, warmth, and erythema, which have not been noted in published ALLEGRO safety analyses. Finally, the duration of symptoms, lasting up to a week in the right shoulder and recurring over several months before resolution, contrasts with the brief, self-resolving sensory complaints documented in clinical studies. Although myalgia was reported in ALLEGRO as a musculoskeletal adverse event (up to 8.8% in higher-dose groups), the character and persistence of pain in this patient were distinct from the mild, nonspecific myalgias noted in trial populations [22].

Implications

Reported neurological adverse events, such as paresthesia or dysesthesia, were uncommon, generally mild in severity [23]. Taken together, these findings suggest that the neuropathic-like pain described in this patient is unusual, temporally associated with ritlecitinib, but not yet supported by broader human safety data. This highlights both the uncertainty of causality and the importance of continued pharmacovigilance and case reporting to further clarify the long-term neurological safety of JAK inhibitors.

Deuruxolitinib safety considerations

Deuruxolitinib represents a promising therapeutic option for severe AA; however, emerging evidence suggests that it may carry a higher burden of adverse events compared with other JAK inhibitors. In a recent systematic review and network meta-analysis, Yan et al reported that deuruxolitinib was associated with an overall greater likelihood of treatment-related adverse events [17]. Qi and Li further demonstrated a significantly increased probability of acne and elevated creatine phosphokinase (CPK) levels, with a clear dose-dependent relationship [24]. CPK elevation is particularly important given its potential association with muscle injury or subclinical myopathy, underscoring the need for routine laboratory monitoring during therapy.

Additional adverse events documented in clinical studies include upper respiratory tract infections, headache, nausea, and laboratory abnormalities such as lipid elevations and liver enzyme changes [25]. While most events are reported as mild to moderate, the higher frequency of acneiform eruptions and CPK abnormalities relative to other JAK inhibitors suggests that dermatologic and musculoskeletal monitoring should be prioritized. Importantly, deuruxolitinib is metabolized via CYP2C9, and current prescribing guidance recommends avoidance altogether in poor metabolizers [25]. This highlights the role of pharmacogenomic testing in tailoring therapy.

Taken together, these safety considerations emphasize the need for individualized treatment planning in patients with refractory AU. Beyond pharmacogenomic tailoring, clinicians should maintain close vigilance for dermatologic side effects, laboratory abnormalities (particularly CPK elevations), and metabolic changes during treatment. Continued post-marketing surveillance and long-term studies will be essential to better define the safety profile of deuruxolitinib and to determine whether its higher adverse event burden ultimately limits its therapeutic benefit in certain patient populations.

Defining treatment failure in the context of published efficacy data

For this discussion, SALT refers to the Severity of Alopecia Tool. SALT is a validated numerical scale (0-100) that quantifies the extent of scalp hair loss in AA, with 0 indicating no hair loss and 100 representing complete scalp hair loss. The score is calculated by estimating the percentage of hair loss in four scalp regions (vertex, right profile, left profile, occiput), each weighted by its respective surface area, to generate a standardized measure of overall disease severity. SALT is widely used in clinical trials and longitudinal studies to objectively monitor treatment response and disease progression.

Given that SALT is widely used to monitor therapeutic efficacy, we defined JAK inhibitor “failure” in this case as the absence of clinically meaningful hair regrowth after an adequate trial period (≥6 months) or the discontinuation of therapy due to adverse effects. To contextualize these definitions of treatment failure, it is important to review clinical trial and real-world evidence evaluating the efficacy of JAK inhibitors in alopecia areata.

In a single-center retrospective, observational cohort study that addressed a literature gap on the effects of tofacitinib on a large cohort of AA patients in "long-term real-world practice", 126 patients with AA were treated with tofacitinib between February 2021 and December 2022, and tofacitinib has shown meaningful efficacy. Additionally, in another real-world "drug survival" cohort with tofacitinib, ~33.8% of patients responded by 24 weeks [26]. 

Although not used in this patient, baricitinib has demonstrated significant efficacy in phase III trials. In two pivotal studies, approximately 35-40% of patients achieved SALT ≤ 20 at 52 weeks, with responses sustained through 104 weeks [27]. In the BRAVE-AA1 and BRAVE-AA2 randomized, placebo-controlled phase III trials, patients receiving baricitinib 4 mg daily had significantly higher rates of hair regrowth compared to placebo by week 36 [28]. Similarly, in a Japanese clinical trial of 17 patients treated with uninterrupted 4 mg daily dosing, SALT20 responses were achieved in 29.4% at week 24, 52.9% at week 36, 58.9% at week 48, and 64.7% at week 60 [29].

Ritlecitinib has also shown efficacy in AA. In trials, a subset of patients achieved substantial regrowth over 24 weeks and beyond [30]. Hair regrowth was sustained through week 48 in patients with response at week 24. Up to one-third of patients who did not meet the target efficacy at week 24 achieved response with continued ritlecitinib treatment [30].

Deuruxolitinib is an oral JAK1/JAK2 inhibitor. After 24 weeks of treatment with Deuruxolitinib 12 mg twice daily, 41.3% of patients achieved SALT ≤20, significantly higher than the control group (0.8%), and it was better than Ritlecitinib 50 mg and Baricitinib 4 mg in patients with severe AA [27].

In contrast, our patient failed to derive meaningful benefit from the systemic JAK inhibitors he received, highlighting the extraordinary refractoriness of his disease. Because the patient did not receive ruxolitinib (topical or systemic), we do not discuss its efficacy or penetration here. Baricitinib is included for comparative purposes only, as it was not part of this patient’s treatment regimen.

## Conclusions

This case illustrates the complexity of managing refractory AU, particularly in the context of polyautoimmunity and early-onset disease. Despite advances in understanding AA pathogenesis and the introduction of JAK inhibitors, therapeutic success remains inconsistent, and potential adverse events may emerge. Personalized approaches, including pharmacogenomic profiling and vigilant monitoring for rare side effects, are essential for optimizing outcomes. This case underscores the need for careful long-term monitoring of efficacy, safety, and quality-of-life outcomes in patients receiving targeted therapies. Continued research into the genetic, immunologic, and environmental determinants of AU will be critical in advancing individualized, durable treatment strategies. Finally, we report an unusual clinical observation of episodic neuropathic-like musculoskeletal pain that arose during ritlecitinib therapy. While the temporal association suggests a possible adverse drug reaction, causality cannot be firmly established due to the absence of a comprehensive neurological evaluation. This case underscores the importance of further studies and case reports to better characterize the neurological safety of JAK inhibitors.
